# Changes in severity of problem gambling and subsequent suicide attempts: a longitudinal survey of young adults in Great Britain, 2018–20

**DOI:** 10.1016/S2468-2667(23)00008-7

**Published:** 2023-03-01

**Authors:** Heather Wardle, Viktorija Kesaite, Sarah Tipping, Sally McManus

**Affiliations:** School of Social and Political Sciences, University of Glasgow, Glasgow, UK; Faculty of Public Health and Policy, London School of Hygiene & Tropical Medicine, London, UK; School of Social and Political Sciences, University of Glasgow, Glasgow, UK; Sarah Tipping Consultant, Lingfield, UK; School of Health and Psychological Sciences, City, University of London, London, UK

## Abstract

**Background:**

Cross-sectional studies identify problem gambling as a risk factor for suicidality. Using an online longitudinal survey, we aimed to examine the association between changes in severity of gambling behaviour and attempted suicide.

**Methods:**

The Emerging Adults Gambling Survey is a longitudinal survey of people in England, Scotland, and Wales, aged 16–24 years interviewed online between June 25 and Aug 16, 2019 (wave 1) and 1 year later between July 13 and Oct 8, 2020 (wave 2). The Problem Gambling Severity Index (PGSI) was administered at both waves. Multivariable logistic regression models examined wave 1 PGSI score and between-wave change in PGSI score as risk factors for suicide attempts at wave 2, unadjusted and with adjustment for wellbeing, anxiousness, impulsivity, perceived loneliness, and suicide attempts at wave 1.

**Findings:**

3549 participants were interviewed in wave 1 and 2094 were interviewed in wave 2, of whom 1941 were included in this analysis (749 [39%] men; 1192 women [61%]). Prevalence of attempted suicide did not change between waves (wave 1: 3·7% [95% CI 2·9–4·8], n=75; wave 2: 3·3% [2·5–4·3], n=65). 78·9% (95% CI 76·7–80·9, n=1575) of participants had stable PGSI scores between the two waves, 13·7% (11·9–15·6, n=233) of participants had a decrease in PGSI score by 1 or more, and 7·5% (6·2–8·9, n=133) had an increase in PGSI score by 1 or more. An increase in PGSI scores over time was associated with suicide attempt at wave 2, even with adjustment for baseline PGSI score and other factors (adjusted odds ratio 2·74 [95% CI 1·20–6·27]). Wave 1 PGSI score alone was not associated with suicide attempt at wave 2 in fully adjusted models.

**Interpretation:**

Repeated routine screening for changes in gambling harm could be embedded in health, social care, and public service settings to allow effective identification and suicide prevention activities among young adults.

**Funding:**

Wellcome Trust.

## Introduction

Suicide is a leading cause of death among young adults, especially young men.^1^ In recent years, increased attention has been given to the extent to which gambling should be considered a risk factor for suicidality.^2^ In 2022, the Department of Health and Social Care in England launched a call for evidence relating to mental health and wellbeing to inform their revised Suicide Prevention Strategy.^3^ They noted the association between gambling disorder and suicidality but highlighted the need for longitudinal evidence on causal mechanisms and pathways.^3^ Similarly, evidence reviews have concluded that there is a relationship between problem gambling severity and suicidality, including self-harm,^4^ but also highlighted substantial limitations in the quality of the evidence produced, with a reliance on cross-sectional studies.^4^

Those with lived experience of gambling harms often cite suicidality, both ideation and attempts, as a major feature of their experience.^5^ Despite this, debate continues regarding the extent to which gambling behaviour should be considered a risk factor for suicidality or whether these associations might be better explained by other simultaneously occurring factors, such as depression, or co-exist as part of a complex nexus of issues.^6^

Few longitudinal studies exist that have examined the relationship between gambling disorder and suicidality, and fewer still have examined this relationship among young people. Studies of either the general population or those with gambling disorder that incorporate a longitudinal element have noted a relationship between gambling disorder and increased risk of suicide mortality or increased risk of suicide attempts.^7–9^

A focus on young people is needed given concurrent heightened risk of both suicidality and gambling disorder among this age cohort.^1,10^ Those aged 18–24 years, sometimes termed emerging adults, are also likely to have a greater propensity for risk-taking behaviour, including impulsivity and engaging in sensation-seeking experimentation before settling into adult roles and responsibilities.^11^ Arguably, the need to examine these associations for young adults has become more evident since the COVID-19 pandemic, when young people experienced substantial disruption to usual life transitions and because, in Great Britain (England, Scotland, and Wales), gambling disorders are typically higher for this age group.^12^

This study aims to contribute to this evidence base by examining the extent to which changes in problem gambling severity preceded a suicide attempt, using a longitudinal sample of young adults in Great Britain. A previous study examined this relationship cross-sectionally, finding that problem gambling was associated with suicide attempts among both young men and young women, and that this association persisted after adjusting for anxiousness, impulsivity, life satisfaction, and other factors.^13^ Building on this previous study, the same group of young men and women were re-interviewed 1 year later. The aim of the current analysis was to explore how changes in problem gambling severity in the previous year predicted subsequent suicide attempts as the main outcome and suicidal thoughts in a supplementary analysis, while controlling for the same range of covariates.

## Methods

### Study design and participants

The Emerging Adults Gambling Survey is a longitudinal survey of people aged 16–24 years living in Great Britain. Participants were first interviewed online between June 25 and Aug 16, 2019 (wave 1) and were re-interviewed online 1 year later (July 13 to Oct 8, 2020; wave 2).^14^ Non-response was examined by a range of factors, and biases resulting from this attrition were addressed with the development of study-specific longitudinal weights (see appendix pp 3–4 for details). Participants who did not answer the questions on suicide attempts were excluded from analysis (see appendix p 5 for details of treatment of missing data).

Participants were drawn from YouGov’s online panel of more than 1 million people living in Great Britain. Those aged 16–24 years, who had not taken part in any other YouGov study on gambling in the past year were eligible to take part. YouGov sent email invitations to eligible panellists, without advertising the survey’s content, in which participants were invited to click through to the bespoke survey. The first page of the initial wave of the bespoke survey described our aims and objectives, advised participants that we would recontact them 1 year later, and obtained written informed consent. In wave 1, 93% of people who accessed this page went on to complete the survey. Participants received YouGov points (equivalent to £0·5 in value) for taking part.

The questionnaires for wave 1 and wave 2 covered gambling, gaming, social media use, health-related behaviours, suicidal thoughts, and suicidal attempts; it was developed by HW and reviewed by an expert panel. In both waves, the first 250 responses from mainstage data collection were reviewed for consistency, routing accuracy, and to establish timing thresholds for seriousness checks.

The study protocol was registered^14^ and ethics approval for the study was granted by the London School of Hygiene & Tropical Medicine’s Ethics Review Panel (reference 16023). The authors assert that all procedures contributing to this work comply with the ethical standards of the relevant national and institutional committees on human experimentation and with the Helsinki Declaration of 1975, as revised in 2008.

### Measures and outcomes

The primary outcome was having made a suicide attempt in the 12 months before wave 2. We focused our primary analysis on suicide attempts because these are most important in relation to suicide and suicide prevention. However, supplementary analysis repeats our methodological approach using past year suicidal thoughts as the outcome (appendix pp 7–8).

The question about suicide attempts was adapted from the Adult Psychiatric Morbidity Survey^15^ and asked “In the last 12 months, have you ever made an attempt to take your life, by taking an overdose of tablets or in some other way?” The same wording was used in both waves of data collection.

In both waves, participants who had gambled (including on lotteries) in the past year completed the Problem Gambling Severity Index (PGSI), a validated tool for the identification of gambling problems^16^ (wave 1 α=0·79; wave 2 α=0·79). The PGSI comprises nine items, with responses coded on a four-point scale ranging from “never” (0) to “almost always” (3). In each wave, a total PGSI score ranging from 0 to 27 was produced, where those who had not gambled were assigned a score of 0. Our analysis used PGSI score at wave 1 and changes in PGSI scores between waves. PGSI scores at wave 1 were compared with PGSI scores at wave 2 and coded as follows: no change in PGSI scores between waves; PGSI score increased by 1 or more; PGSI score decreased by 1 or more.

The adjustment variables of impulsivity, personal wellbeing, risky alcohol consumption, perceived loneliness, video game use and social media use, ethnicity, age, local-area-level deprivation, parental academic attainment, economic activity, and suicide attempts at wave 1 were all measured at baseline (wave 1). These variables were chosen on the basis of known association with either experience of problem gambling or suicide attempts and to replicate the range of controls used in our previous study.^14^

Impulsivity was measured at wave 1 using a shortened form of the Eysenck Impulsivity Scale validated for use among adolescents.^17–19^ Responses to seven statements relating to impulsivity were recorded on a five-point scale with response options ranging from very true (1) to not at all true (5; α=0·87). Impulsivity scores were computed as the mean of the seven questions (wave 1 mean 2·28 [SD 0·87]).

Personal wellbeing was captured using the harmonised UK Office for National Statistics four-item measure of personal wellbeing.^20^ Participants rated their current levels of life satisfaction, whether they do things that they feel are worthwhile, how happy they felt yesterday, and how anxious they felt yesterday on a scale of 0 to 10.

Risky alcohol consumption was identified using the Modified Single Alcohol Screening Questionnaire (M SASQ).^21^ The M SASQ uses one item from the Alcohol Use Disorders Identification Test about frequency of consuming eight or more units of alcohol for men or six more units of alcohol for women in a single event in the past year. This, combined with a question on past year alcohol consumption, produces the following categories: non-drinking, an M SASQ score of 0–2 (never or rarely consumes eight or six units of alcohol on a single occasion), and a M SASQ score of 3 or more (consumes eight or six units of alcohol or more on a single occasion at least weekly). A score of three or more identifies drinkers at higher risk.

One item from the Social Functioning Questionnaire assessed perceived loneliness.^22^ Participants were asked to rate the extent to which they had felt “lonely and isolated from other people” in the previous 2 weeks on a four-point scale: very much, sometimes, not often, and not at all. This was grouped into a binary variable representing those experiencing “very much” loneliness versus those experiencing loneliness not at all, not often, or sometimes.

Participants were asked how often they had played video games in the past year, coded into those who played video games at least weekly and those who played video games less than weekly (including never). Participants were asked how much time they spent on social media on a usual day, with response options ranging from less than half an hour to 7 or more hours a day (grouped into 5 or more hours per day because of base sizes).

Ethnicity was reported in wave 1 using the UK’s Office of National Statistics harmonised ethnic group question. Because of low base sizes, responses were grouped into White, Asian, Black, and mixed ethnic group or other. Age at wave 1 was captured in single-year ages and used as a continuous measure. Local-area-level deprivation was measured using English, Scottish, and Welsh Indices of Multiple Deprivation scores matched at the output area and quintiled for analysis. Participants reported the academic attainment of their parents with responses grouped by whether at least one parent had a degree or higher or whether both parent’s qualifications were lower than degree level. Participants were asked to report their economic activity, coded as whether they were in education, employment, or training, or not.

### Statistical analysis

Analyses reported here represent secondary analysis of an existing dataset. As such, specific power calculations for these analyses were not produced. Details of power calculations for the original study and its primary outcome are given in the appendix (p 6).

Weighted frequencies described the extent of change in PGSI scores and the characteristics of the sample (see appendix pp 3–4 for weighting procedures). Unadjusted binary logistic regression examined the extent to which changes in PGSI scores, PGSI score (at wave 1), and each control variable were associated with reporting suicide attempts at wave 2.

Because the number of cases within the dependent variable was low (n=65), we identified a parsimonious set of control variables. This was first informed by knowledge of factors likely to be associated with suicide attempts and variables used in the previous study^13^ and the unadjusted regression results. Thus, variables significant at the 5% level in the unadjusted logistic regression models were entered into a multivariable logistic regression model, with suicide attempts at wave 2 as the dependent variable and PGSI score at wave 1, PGSI score change, and both mutually adjusted as the main independent variables. All control variables measured behaviours and experiences at wave 1 and were treated as fixed.

Apart from impulsivity, PGSI score at wave 1, and personal wellbeing variables, which were entered into models as continuous variables, all control variables were categorical. The linearity of all continuous variables against the logit function was confirmed using the linktest command in Stata (version 15).

Collinearity was examined by calculating the variance inflation factors of all independent variables. With the exception of happiness, life satisfaction, and whether someone felt their life had meaning, all independent variables had variance inflation factor values of less than 2, indicating they were not too closely correlated.^23^ Variance inflation factor values showed that three of the four wellbeing measures (happiness, wellbeing, and meaning) were closely correlated. Only one of these three variables, in addition to anxiousness, was included in the final model, chosen on the basis of strength of association with the primary outcome measure. Sensitivity tests were conducted by including each of the excluded wellbeing measures individually in the main models. This involved re-running the fully adjusted models, with wellbeing and meaning entered individually, to assess effect on results. Other sensitivity tests were performed by splitting data at random into a sample representing 75% of the original sample (rather than halves, because of base sizes) and repeating the fully adjusted models. In addition, the fully adjusted models were repeated omitting suicide attempt status at wave 1 (appendix p 10). All analyses were performed using the complex survey function in Stata (version 15) with weights to adjust for attrition (appendix pp 3–4). This produced a Wald’s F-test as the default test of significance.^24^ In tables showing frequencies, true (unweighted) bases and sample sizes are presented while the analyses are adjusted using attrition weights.

### Role of the funding source

The funder of the study had no role in study design, data collection, data analysis, data interpretation, or writing of the report.

## Results

3549 participants were interviewed in wave 1 and 2094 were interviewed in wave 2. In wave 2, 14 participants, who completed the survey in less than 1 SD of the mean completion time (less than 2 min 30 s for non-gamblers and less than 4 min for gamblers), were removed, giving a final wave 2 sample size of 2080. This represents a retention rate between waves 1 and 2 of 58·6%. A further 139 participants did not answer the questions on suicide attempts and were excluded from the analysis. Thus, data from 1941 wave 2 participants (749 [39%] men and 1192 women [61%]; 1642 [85%] White, 76 [4%] mixed ethnicity or other ethnic group, 112 [6%] Asian, 35 [2%] Black, and 76 [4%] missing ethnicity data) were used in this analysis.

Weighted prevalence of having made a suicide attempt in the past year was similar between wave 1 and wave 2, with 75 (3·7% [95% CI 2·9–4·8]) participants reporting having made a suicide attempt at wave 1 and 65 (3·3% [2·5–4·3]) reporting this at wave 2. 20 (1·0% [95% CI 0·6–1·5]) of these participants reported suicide attempts in the previous 12 months at both wave 1 and wave 2 (table 1).

Overall, PGSI scores remained similar for most people between waves, with 1575 (78·9% [95% CI 76·7–80·9]) participants reporting the same PGSI score in wave 1 and wave 2 (table 1). Of these participants, the majority had a PGSI score of 0, indicating that they were either non-gamblers or gambled with no reported difficulties in both waves. However, 233 (13·7% [95% CI 11·9–15·6]) participants had a reduction of at least 1 in their PGSI score between waves 1 and 2, and 43 (2·8% [2·0–3·8]) participants had a reduction of 5 or more in their PGSI score (table 1). Among those with a reduced PGSI score, the mean reduction was 3·8 (SD 4·4). 133 (7·5% [95% CI 6·2–8·9]) participants had an increase of at least 1 in their PGSI score between waves 1 and 2, with 31 (2·0% [1·3–2·8]) participants having an increase of 5 or more (table 1). Among those with an increase in PGSI score, the mean increase was 4·4 (SD 4·7).

Unadjusted binary logistic regression showed that the odds of reporting a suicide attempt at wave 2 were 4·49 (95% CI 2·17–9·29) times higher among those whose PGSI score increased by 1 or more than those whose PGSI score remained the same (table 2). Additionally, the odds ratio (OR) for suicide attempts was 1·11 (1·05–1·17) for every unit higher PGSI score at wave 1 (table 2). Impulsivity and anxiousness at wave 1 were positively associated with reporting a suicide attempt at wave 2 (table 2). Happiness was inversely associated with reporting a suicide attempt. Participants who felt lonely “very much” at wave 1 had an OR for suicide attempts at wave 2 of 3·55 (95% CI 2·01–6·26) compared with those who perceived loneliness sometimes or less. Previous suicide attempts were strongly associated with suicide attempts at wave 2, with an OR of 14·6 (95% CI 7·6–28·2) among those who attempted suicide previously compared with those who had not attempted suicide at wave 1 (table 2). These control variables were all included in the fully adjusted model.

Unadjusted models did not show evidence of an association between age, gender, ethnicity, employment, deprivation, parental qualifications, alcohol consumption, social media use, or video game use and suicide attempts at wave 2.

Results for the three multivariable logistic regressions, including a standardised set of controls, are shown in table 3. The first assessed the association between wave 1 PGSI score and suicide attempts. The second assessed the association between changes in PGSI score and suicide attempts at wave 2, and the third examined changes in PGSI score while also controlling for PGSI score at wave 1.

There was no association between PGSI score at wave 1 and subsequent suicide attempts when impulsivity, happiness, anxiousness, perceived loneliness, and previous suicide attempts were taken into account (table 3). However, there was a significant association between change in PGSI score and suicide attempts when these factors were considered. The OR for a suicide attempt at wave 2 was 2·94 (95% CI 1·36–6·37) among those whose PGSI scores increased compared with those who had no change in scores (table 3). Change in PGSI score was significantly associated with suicide attempts at wave 2 even when PGSI score at wave 1 was controlled for. The OR of suicide attempts among those whose PGSI score increased was 2·74 (95% CI 1·20–6·27) compared with those who had no change in scores.

Sensitivity analysis repeating these models on 75% of the sample, selected at random, showed results of similar magnitude and direction to the results reported in the main analyses (appendix p 9). Supplementary analysis replicating our analytical approach using past-year suicidal thoughts as the outcome showed that PGSI change score was not associated with suicidal thoughts, although wave 1 PGSI score was in the adjusted model, which did not also include changes in PGSI scores. Adjusted odds of suicidal thoughts at wave 2 increased by 1·05 for each increase in PGSI score at wave 1 (appendix p 8). Further sensitivity tests checking the effect of using any one of the three correlated wellbeing measures gave similar results to those reported here (data available on request).

## Discussion

In our study, among young adults, an increase in severity of problematic gambling was associated with making a suicide attempt. These results attenuated but remained significant in models that took into account impulsivity, loneliness, low wellbeing, and anxiousness. These factors have previously been found to increase the likelihood of both suicide and problem gambling and have been postulated as alternative explanations for the observed association between suicide attempts and problem gambling severity in cross-sectional reports.^13,25,26^ Our data show that although these factors affect the strength of the association between suicide attempts and PGSI score, they do not account for it in full. Notably, PGSI severity was associated with suicide attempts irrespective of someone’s baseline PGSI score, suggesting that increasing PGSI scores, regardless of their original starting point on the PGSI continuum, are associated with an increased risk of suicide attempts. To our knowledge, this is one of the first studies to identify this pattern among young people, although findings are consistent with retrospective reports from those with lived experience of harms.^5^ Supplementary analyses also indicated that PGSI score at wave 1 was associated with suicidal thoughts at wave 2, but PGSI change scores were not. This requires further investigation.

Our study showed that gambling behaviours are dynamic and change over time, with evidence of both increases and decreases in PGSI scores. Indeed, more people in our sample showed a decrease in PGSI score than an increase, although this might be related to restrictions in gambling supply during the COVID-19 pandemic.^27,28^ Future research could explore the extent to which a dose–response relationship between changing PGSI scores and suicide attempts might be evident, with greater increases in PGSI scores associated with greater risk of suicide attempts (our study was underpowered to examine this).

These findings support a growing body of evidence demonstrating the severity of harms associated with gambling, requiring a strong regulatory and legislative response. They also raise important questions about preferred methods for intervening with individuals deemed to be at risk. Internationally, governments are increasingly requiring gambling operators to perform risk analyses of customer’s data to identify those at increased risk of gambling harms. This theoretically includes identifying those whose gambling severity is increasing. Once customers at higher risk have been identified, regulators typically require gambling operators to intervene. What these interventions entail is unclear, and their efficacy is subject to little independent evaluation. Regulators should question whether customer service staff within gambling companies, working to a range of commercial objectives not just the prevention of harms, are best placed to perform customer interactions when there might be an elevated risk of suicide. Both the ethics and efficacy of this approach should be re-evaluated. Should regulators retain this requirement, all staff engaging in customer interactions could be required to have regular, independent, transparent, and robust suicide prevention and intervention training. This could be made a mandatory condition of licensing and could replicate the approach used in reforms to the financial services sector.

Our findings suggest that routine screening of young adults over time is needed to identify those for whom gambling harms are escalating. Primary care and other health, social care, and public services settings should embed routine measures of gambling behaviours into practice to enable this identification.^29^ Recognising the full continuum of gambling behaviours is important and, in terms of identifying those at greatest risk of attempting suicide, any increase along the continuum should be of concern.

There are some limitations of our study to consider. The sample for this study was drawn from a non-probability panel with attendant issues of generalisability. Nevertheless, compared with other sampling frames, it has good sample coverage, including young people both in and out of full-time education (unlike samples drawn from higher education institutes or the postcode address file, which excludes those living in halls of residence). A review of evidence has shown that although online non-probability methods perform poorly when presenting point-based or prevalence estimates, they can perform better (although still with some issues) when measuring association between variables,^30^ as this study does. Attrition between the two survey waves in our study was high at 41·4%, but this is commensurate with other longitudinal studies of young people. Although rates of attrition were broadly similar between those who had attempted suicide at wave 1 and those who had not (appendix p 4), it is uncertain whether those who had attempted suicide at wave 1 and responded were systematically different to those who had attempted suicide at wave 1 and dropped out. Base sizes were too small to examine this difference but because attrition might disproportionately affect those with less stable or deteriorating circumstances, the results reported here might be conservative.

Suicide attempts in the past year were rare but broadly commensurate with prevalence estimates from probability sample surveys, such as the Adult Psychiatric Morbidity Survey 2014.^25^ This rarity limited the number of controls we could include in the fully adjusted model and non-association might be due to the small sample size. Furthermore, the study was not originally designed to focus on suicide attempts but rather to look at gambling behaviour changes over time. Some covariates such as physical health, depression, or experience of childhood trauma were not included in the study, which could further explain the relationship between problem gambling and suicide attempts. It is plausible that increasing difficulties with gambling do lead to increases in attempted suicide. It is also plausible that depression or other factors play a role in both. This needs further examination. Regarding control variables (such as impulsivity and wellbeing), change between survey waves was not taken into account, and such change could have influenced the outcome variable. Similarly, the analyses could not account for the change in suicide behaviour between the two waves. Future studies could address these issues and include more detailed measures of personal wellbeing, which were only represented in this analysis by two single item questions because of covariance. Furthermore, non-gamblers were not separated from non-problem gamblers, which might have a bias on the observed effects. Because of small sample sizes, we were unable to examine how these patterns varied for men and women, or for those of different ethnic backgrounds, and whether a suicide attempt at wave 2 was associated with someone being classified as experiencing problem gambling at wave 1 (ie, had a PGSI score of 8 or more). Within our sample, both of these were statistically rare, rendering analysis of this nature unstable. Instead, we included PGSI score at wave 1 within our analysis.

Our study showed that among young adults living in Great Britain, increasing PGSI scores were associated with greater risk of suicide attempts. This was evident irrespective of confounders and previous PGSI scores, suggesting that any increase in severity for this age group along the continuum of gambling harms might be an important marker warranting early investigation. Future studies should explore this further, examining results among all adults, and among samples using random probability designs. Nevertheless, these findings suggest that, in addition to calls for more universal preventive action, routine and repeated screening for gambling harms could be embedded within relevant health, social care, and public service settings to allow effective identification and suicide prevention activities among young adults at elevated risk.

## Supplementary Material

Supplementary Appendix

## Figures and Tables

**Table 1:** Changes in PGSI score and suicide attempts between 2018 and 2019 (wave 1) and between 2019 and 2020 (wave 2)

	Number of participants (n=1941)
**PGSI score**	
PGSI score decreased by 5 or more	43 (2·8%; 2·0–3·8)
PGSI score decreased by 4	10 (0·7%; 0·4–1·3)
PGSI score decreased by 3	21 (1·4%; 0·9–2·2)
PGSI score decreased by 2	40 (2·4%; 1·7–3·4)
PGSI score decreased by 1	119 (6·4%; 5·2–7·8)
No change in PGSI score	1575 (78·9%; 76·7–80·9)
PGSI score increased by 1	56 (3·1%; 2·3–4·1)
PGSI score increased by 2	23 (1·2%; 0·7–1·8)
PGSI score increased by 3	17 (0·9%; 0·5–1·5)
PGSI score increased by 4	6 (0·4%; 0·2–1·0)
PGSI score increased by 5 or more	31 (2·0%; 1·3–2·8)
**Suicide attempt in past 12 months**	
Suicide attempts in neither 2018–19 or 2019–20	1821 (94·0%; 92·7–95·0)
Suicide attempts reported in 2018–19 only	55 (2·8%; 2·1–3·7)
Suicide attempts reported in 2019–20 only	45 (2·3%; 1·7–3·2)
Suicide attempts reported in 2018–19 and 2019–20	20 (1·0%; 0·6–1·6)

Data are n (%; 95% CI). All percentage estimates have survey weights applied, while n represents the absolute unweighted number. PGSI=Problem Gambling Severity Index.

**Table 2:** Unadjusted ORs for suicide attempts at wave 2

	Proportion of participants (95% CI) or mean (SD; 95% CI)	Total number of participants	Number of participants who reported attempted suicide at wave 2	Unadjusted OR (95% CI)	p value
Change in PGSI score					
No change in PGSI score	78·9% (76·7–80·9)	1575	41	1 (ref)	<0·0001
PGSI score increased	7·5% (6·2–8·9)	133	11	4·49 (2·17–9·29)	¨
PGSI score decreased	13·7% (11·9–15·6)	233	11	1·78 (0·84–3·79)	¨
PGSI score at wave 1	0·8 (3·1; 0·7–1·0)	1941	65	1·11 (1·05–1·17)	<0·0001
Gender at wave 1					
Male	53·2% (50·8–55·6)	749	22	1 (ref)	0·34
Female	46·8% (44·4–49·3)	1192	43	1·31 (0·75–2·28)	¨
Age in single years at wave 1	20·1 (2·4; 20·0–20·3)	1941	65	0·99 (0·89–1·12)	0·92
Ethnicity at wave 1					
White	83·6% (81·6–85·4)	1642	56	1 (ref)	0·45
Mixed-race or other	4·4% (3·5–5·7)	76	1	0·25 (0·03–1·85)	¨
Asian	6·5% (5·3–7·9)	112	2	0·74 (0·16–3·39)	¨
Black	1·4% (1·0–2·1)	35	1	0·70 (0·09–5·29)	¨
Missing	4·1% (3·2–5·1)	76	5	1·82 (0·67–4·91)	¨
Employment status at wave 1					
Employed, in education, or in training	88·8% (82·7–90·2)	1698	55	1 (ref)	0·45
Not in employment, education, or training	11·2% (9·8–12·8)	243	10	1·33 (0·63–2·82)	¨
Area deprivation at wave 1					
Not living in most deprived quintile	67·5% (65·0–69·9)	1424	44	1 (ref)	0·27
Living in most deprived quintile	18·1% (16·3–20·1)	361	16	1·62 (0·85–3·07)	¨
Missing	14·4% (12·4–16·7)	156	5	0·81 (0·30–2·21)	¨
Parents’ qualifications at wave 1					
Degree or higher	58·6% (56·2–61·1)	1138	32	1 (ref)	0·42
Lower than degree or none	36·6% (34·2–39·0)	707	28	1·34 (0·76–2·37)	¨
Missing	4·8% (3·9–6·0)	96	5	1·77 (0·63–4·94)	
Alcohol status at wave 1					
Non-drinking	27·2% (25·0–29·5)	496	17	1 (ref)	0·35
M SASQ score 0–2	59·8% (57·3–62·2)	1176	34	0·87 (0·46–1·65)	¨
M SASQ score 3 or more (higher risk drinking)	13·1% (11·6–14·8)	269	14	1·47 (0·67–3·24)	¨
Impulsivity score at wave 1	2·3 (0·9; 2·2–2·3)	1941	65	2·09 (1·55–2·84)	<0·0001
Social media use at wave 1					
Less than 1 h per day	12·7% (12·7–14·5)	248	5	1 (ref)	0·65
1 h per day to <2 h per day	19·8% (17·9–21·9)	371	12	1·56 (0·50–4·90)	¨
2 h per day to <3 h per day	21·1% (19·1–23·2)	411	12	1·13 (0·36–3·56)	¨
3 h per day to <5 h per day	19·2% (17·4–21·3)	376	12	1·25 (0·40–3·95)	¨
≥5 h per day	27·1% (25·0–29·5)	535	24	1·84 (0·64–5·27)	¨
Video game play at wave 1					
Does not play video games on weekly basis	47·6% (45·1–50·1)	1010	36	1 (ref)	0·57
Plays video games once a week or more	52·4% (49·9–54·9)	931	20	0·85 (0·49–1·47)	¨
Perceived loneliness at wave 1					
Not at all, not often, or sometimes	86·1% (84·3–87·7)	1671	39	1 (ref)	<0·0001
Very much	13·9% (12·3–15·8)	270	26	3·55 (2·01–6·26)	¨
Happiness score at wave 1^*^	6·5 (2·3; 6·4–6·6)	1941	65	0·79 (0·69–0·89)	<0·0001
Anxiousness score at wave 1†	4·5 (2·8; 4·4–4·6)	1941	65	1·33 (1·18–1·50)	<0·0001
Suicide attempts at wave 1					
No	96·3% (95·2–97·1)	1866	45	1 (ref)	<0·0001
Yes	3·7% (2·9–4·8)	75	20	14·61 (7·57–28·23)	¨

All percentage estimates have survey weights applied, while n represents the absolute unweighted number. M SASQ=Modified Single Alcohol Screening Questionnaire. OR=odds ratio. PGSI=Problem Gambling Severity Index.

* Higher happiness scores indicate higher happiness.

† Higher anxiousness scores indicate higher anxiousness.

**Table 3:** Adjusted ORs for suicide attempts at wave 2

	Model 1: PGSI score at wave 1		Model 2: PGSI change score		Model 3: PGSI score at wave 1 and PGSI change score	
	Adjusted OR (95% CI)	p value	Adjusted OR (95% CI)	p value	Adjusted OR (95% CI)	p value
PGSI score at wave 1	1·04 (0·98–1·10)	0·23	NA	NA	1·03 (0·95–1·12)	0·42
Change in PGSI score						
No change in PGSI score	NA	NA	1 (ref)	0·024	1 (ref)	0·037
PGSI scores increased	NA	¨	2·94 (1·36–6·37)	¨	2·74 (1·20–6·27)	¨
PGSI scores decreased	NA	¨	1·24 (0·59–2·60)	¨	0·98 (0·35–2·77)	¨
Perceived loneliness at wave 1						
Not at All, not often, or sometimes	1 (ref)	0·87	1 (ref)	0·86	1 (ref)	0·91
Very much	1·06 (0·51–2·22)	¨	1·07 (0·50–2·28)	¨	1·04 (0·50–2·20)	¨
Impulsivity score at wave 1	1·48 (1·09–1·99)	0·011	1·50 (1·15–1·96)	0·0030	1·45 (1·09–1·94)	0·011
Happiness score at wave 1^*^	0·85 (0·73–0·98)	0·023	0·85 (0·74–0·98)	0·022	0·84 (0·72–0·97)	0·016
Anxiousness score at wave 1†	1·14 (1·01–1·28)	0·036	1·13 (1·01–1·27)	0·030	1·13 (1·00–1·27)	0·047
Suicide attempts at wave 1						
No	1 (ref)	<0·0001	1 (ref)	<0·0001	1 (ref)	<0·0001
Yes	6·27 (2·90–13·53)	¨	6·06 (2·74–13·38)	¨	5·64 (2·57–12·39)	¨

Model fit using Hosmer-Lemeshow goodness of fit test was F-statistic (9, 1932) 0·90 (Prob>F 0·52) for PGSI score at wave 1, 0·4O (Prob>F 0·94) for PGSI change score, and 0·67 (Prob>F 0·74) for PGSI change additionAlly adjusted for PGSI score at wave 1. OR=odds ratio. PGSI=Problem Gambling Severity Index.

* Higher happiness scores indicate higher happiness.

† Higher anxiousness scores indicate higher anxiousness.

## Data Availability

Data will be deposited with the UK Data Service archive in 2023 upon completion of the project. Until then, reasonable requests for data access should be submitted to the lead author (HW).

## Research in context

### Evidence before this study

We searched titles and abstracts in PubMed on July 20, 2022, to identify prospective studies with a longitudinal element assessing the relationship between suicidality and gambling (see appendix p 2 for full search terms). Original articles were considered eligible for inclusion if they presented empirical analyses of population-level evidence, with searches limited to English language but with no date restriction. Reviews and single case studies were excluded. Longitudinal evidence is scarce, with just seven studies identified, and with some analysis presented cross-sectionally. There is a gap in the evidence on the extent to which gambling affects suicidal behaviours, with evidence using longitudinal datasets limited to the Netherlands, Sweden, Australia, New Zealand, France, and the USA. Furthermore, existing evidence on this relationship with data collected during the COVID-19 pandemic is scarce.

### Added value of this study

This study provides longitudinal evidence that an increase in severity of problem gambling presents a risk for attempted suicide, irrespective of previous problem gambling scores or status. This approach shows that, among a sample of young adults, problem gambling levels are dynamic at the individual level and require monitoring. Irrespective of initial score, an increase in severity was associated with increased risk of suicide attempts.

### Implications of all the available evidence

Although replication and further studies are required, results suggest that routine and repeated screening for gambling harms could be embedded within primary health care, social care, and other relevant public services to allow effective identification and suicide prevention activities among young adults at elevated risk. Regulators and policy makers should assess whether current approaches relying on the gambling industry to identify and interact with those at increasing risk of gambling harm are appropriate for interventions with young adults at increased risk of attempted suicide.
