# Supplementary material for: Changes in severity of problem gambling and subsequent suicide attempts: a longitudinal survey of young adults in Great Britain, 2018–20
Source: Lancet Public Health. Author manuscript; Available in PMC 2023 Jul 10. (PMC7614739; doi:10.1016/S2468-2667(23)00008-7)
Supplement: Supplementary Appendix [file EMS177456-supplement-Supplementary_Appendix.pdf]

THE LANCET
Public Health

Supplementary appendix

This appendix formed part of the original submission and has been peer reviewed. We post it as supplied by the authors.

Supplement to: Wardle H, Kesaite V, Tipping S, McManus S. Changes in severity of problem gambling and subsequent suicide attempts: a longitudinal survey of young adults in Great Britain, 2018–20. *Lancet Public Health* 2023; **8:** e217–25.

Appendices: Changes in severity of problem gambling and subsequent suicide attempts: a longitudinal survey of young adults in Great Britain, 2018-2020.

Appendix A: Search terms used in the literature review for Research in Context
Appendix B: Generation of longitudinal weights between wave 1 and wave 2 of the Emerging Adults Gambling Survey
Appendix C: Treatment of missing values in analysis
Appendix D: Power calculations for Emerging Adults Gambling Survey
Appendix E: Analysis focusing on suicide attempts.
Appendix F: Sensitivity analysis
Appendix G: STROBE checklist

Appendix A: Search terms used for literature review in Research in Context

"Suicide" OR "Suicidal behave* " OR "Suicide ideation" OR "Suicide plan" OR "Suicide attempt" OR "Non-suicidal self-injury" OR "Parasuicide" OR "Self-injure" OR "Deliberate self-harm" OR "Suicidality" OR "Non-fatal suicidal behav* " AND "Gambl*" OR "wager" OR "Gambling expenditure" OR "Gambl* losses" OR "Gross gambling revenue" OR "Gambling frequency" OR "gambling activities" AND "Longitudinal study" OR "Observational study" OR "Cohort study" OR "Prospective" OR "Retrospective" OR "Follow-up".

Appendix B: generation of longitudinal weights between wave 1 and wave 2 of the Emerging Adults Gambling Survey

Methodology
To generate the longitudinal weights, YouGov built a model that predicts which people are likely to drop out between wave 1 and wave 2. They then upweight people who were more *likely* to have dropped out (based on the predicted value), but did not actually dropout. Below are the steps followed to generate the attrition weights used in this study:

1. Take the wave 1 data, and add an attrition variable that tells us whether this respondent answered wave 2 (coded 0 = answered, 1 = did not answer)
2. Run a logistic regression with "attrition variable" as the dependent variable, and the "candidate variables" as predictor variables (see below for details). We then generate predicted probabilities from the regression modelling
3. Drop cases from the datafile who did not respond at wave 2
4. Divide each predicted value by the mean predicted value after dropping wave 2 cases (for example, if respondent has a predicted probability of 0.375, and the mean predicted probability is 0.25, then 0.375 / 0.25 = 1.5. This is the **attrition weight**
5. Multiply the wave 1 weight (which matched the responding sample in wave 1 to the age, gender, deprivation and regional profile of young people living in Great Britain) by the attrition weight. This will be the wave 2 **longitudinal weight.** This is the weighting variable used in this analysis.

Summary
The logistic modelling conducted in this study is based on these attrition predictor variables collected at wave 1:

**Variable Name**	**Description**
W1sex	Gender, coded men/women.
W1AgeBands	Age (grouped), coded 16-18, 19-21, 22-24 years.
W1ethnicg	Self-reported ethnic identity (grouped), coded White/White British; Mixed; Asian/Asian British; Black/Black British; Other.
W1neet	Whether in Employment, Education or Training, coded yes/no.
W1anystudent	Whether currently in higher education, coded, yes/no.
W1qimd	Area Deprivation Decile, coded from least to most deprived.
W1gamfreq2	Frequency of gambling on any activity, coded more than weekly; about weekly; about fortnightly; about monthly; a couple of times a year; did not gamble.
W1anyacty	Whether gambled on any activity in the past year or no, coded yes/no.
W1ngamyr	Number of gambling activities undertaken in the past year, range from 0 to 15.
W1pgsiprob	Problem Gambling Severity Index status, coded 0 = non-gambler/non-problem gambler; 1-2 low risk gambling; 3-7 moderate risk gambling; 8+ problem gambling.

Four variables were significant in the model result: W1sex (Gender) , W1ethnicg (Ethnicity), W1qimd (Area-deprivation) and W1gamfreq2 (Gambling). The model shows that women were less likely to drop out than men. Participants of Black/Black British origin were less likely to drop out than those from White/White British backgrounds. Those whose area deprivation status was unknown

were more likely to drop out than those where this was categorized. Those who gambled were more likely to drop out than those who did not gamble. This was particularly evident for those gambling fortnightly.

Attrition by suicide attempt status

For the analysis presented in this paper, we examined attrition rates by suicide attempt status at wave 1 (see Table B1 below). This shows that those who reported attempting suicide at wave 1 were no more likely than those who had not reported this to drop out of the survey. Respective attrition rates were 43.5% for those who had attempted suicide at wave 1 and 41.5% for those who had not. Because of small base sizes, we were not able to look at whether the profile of those who had attempted suicide in wave 1 (n=64) but dropped out was systematically different from those who had attempted suicide in wave 1 (n=83) but did not drop out. However, we note that our attrition weights do adjust for age, gender and area deprivation. We have noted this as a potential limitation of our article.

Table B1: Rates of attrition by wave suicide attempt status

**Wave 1 suicide attempt status**	Did not take part in wave 2 N (%)	Took part in wave 2 N (%)
Reported attempted suicide at wave 1	64 (43.5%)	83 (56.5%)
Did not report attempted suicide at wave 1	1403 (41.3%)	1997 (58.7%)
Total	1469 (41.4%)	2080 (58.6%)

X^2 (1, 3549) = 0.291; p=0.590.

Appendix C: Treatment of missing values in analysis

This document sets out the number of missing values for each variable included in the analysis and the treatment applied to those values within the modelling. Analysis is based on those who took part in both waves (n=2080), thus missing values are presented for this group.

**Variable**	**Number of missing cases**	**Treatment of missing cases**
Suicide Attempts Wave 2	139	Excluded from analysis
Age	0	N/A
Gender	0	N/A
Ethnic group	93	Coded to dummy category “ethnicity not known”
Employment status	1	Recoded to modal “in employment, education, training”
Parental education	76	Coded to dummy category “educational status unknown”
Alcohol status	0	N/A
Area Deprivation	156	Coded to dummy category “area deprivation unknown”
Social media	0	N/A
Video games	0	N/A
Impulsivity	0	N/A
Happiness	0	N/A
Anxious	0	N/A
Loneliness	0	N/A
Problem Gambling Severity Index Score	0	N/A

Appendix D: Power calculations for Emerging Adults Gambling Survey.

This data uses in this analysis is from the Emerging Adults Gambling Survey. That original survey was designed to examine individual gambling trajectories over time. Sample size calculations were based on being sufficient to estimate change in gambling prevalence between waves, which was not the endpoint of the current analysis. Assuming a between wave correlation of 0.5, the study was designed to be able to detect changes in problem gambling behaviours of +/- 0.3 percentage points (at 80% power).

Appendix E: Analysis focusing on suicide attempts

In supplementary tables 1 and 2, we repeat the analytical procedures documented in the main manuscript using past year suicidal thoughts as the outcome measure. Following the approach developed for the Adult Psychiatric Morbidity Survey 2007, the following question was asked at both wave 1 and wave 2: "In the last 12 months, have you ever thought of taking your life, even if you would not actually do it?". This was binary coded Yes (1) and No (0). As with suicide attempts, 139 participants did not answer this question and were excluded from analysis. Tables S1 show the unadjusted relationship between PGSI change scores and PGSI score at wave 1 with experience of suicidal thoughts in year prior to the wave 2 interview. Table S2 shows the adjusted relationship, using the same set of controls used in the main analysis, which were also all significantly associated with suicidal thoughts at wave 2, and additionally controlling for gender, which was also significant.

**Supplementary Tables 1: Unadjusted odds ratios for suicidal thoughts at wave 2**			
	PGSI score at wave 1		
	OR	95% CI (lower)	95% CI (upper)
**Problem Gambling Severity Index (PGSI) Score (wave 1)**			p>0.001
PGSI score	1.07	1.03	1.11
**Change in PGSI Score**			p=0.060
No change in PGSI score	Reference		
PGSI scores increased	1.31	0.88	1.96
PGSI scores decreased	1.40	1.02	1.92

Supplementary Tables 2: Adjusted odds ratios for suicidal thoughts at wave 2, adjusted and unadjusted									
	Model 1: PGSI score at wave 1			Model 2: PGSI change score			Model 3: PGSI score (wave 1) and PGSI change score		
	AOR	95% CI (lower)	95% CI (upper)	AOR	95% CI (lower)	95% CI (upper)	AOR	95% CI (lower)	95% CI (upper)
**Problem Gambling Severity Index (PGSI) Score (wave 1)**			p=0.019						p=0.087
PGSI score	1.05	1.01	1.10				1.04	0.99	1.10
**Change in PGSI Score**						p=0.1736			p=0.780
No change in PGSI score				Reference			Reference		
PGSI scores increased				1.21	0.77	1.91	1.12	0.71	1.79
PGSI scores decreased				1.39	0.96	2.01	1.13	0.74	1.74
**Gender**			p=0.007			p=0.008			p=0.006
Male	Reference			Reference			Reference		
Female	1.39	1.09	1.76	1.38	1.09	1.75	1.39	1.10	1.77
**Perceived loneliness (wave 1)**			p=0.002			p=0.002			p=0.002
Not at all/Not often/Sometimes	Reference			Reference			Reference		
Very much	1.71	1.21	2.40	1.74	1.23	2.44	1.71	1.22	2.41
**Impulsivity (wave 1)**			p=0.059			p=0.022			p=0.061
Impulsivity score (wave 1)	1.14	1.00	1.32	1.17	1.02	1.35	1.14	0.99	1.32
**Happiness (wave 1)**			p=0.055			p=0.098			p=0.058
Happiness scores (higher scores, higher happiness)	0.94	0.89	1.00	0.95	0.90	1.01	0.94	0.89	1.00
**Anxiousness (wave 1)**			p=0.337			p=0.231			p=0.340
Anxiousness score (higher scores, higher Anxiousness)	1.02	0.98	1.07	1.03	0.98	1.08	1.02	0.98	1.07
**Suicidal thoughts (wave 1)**			p<0.0001			p<0.0001			p<0.0001
No	Reference			Reference			Reference		
Yes	3.88	3.03	4.97	3.91	3.05	5.02	3.88	3.03	4.98
**Hosmer-Lemeshow Goodness of Fit**									
F-statistic (9, 1920)	0.27			0.52			0.23		
Prob>F	0.9815			0.8574			0.9910		

Appendix F: Sensitivity testing

Supplementary Table 1: Sensitivity testing for adjusted odds ratios for suicide attempts at wave 2: run on 75% of the full sample; model 3			
	AOR	95% CI (lower)	95% CI (upper)
**Change in Problem Gambling Severity Index (PGSI) Score**			p=0.0176
No change in PGSI score	Reference		
PGSI scores increased	3.68	1.47	9.24
PGSI scores decreased	1.20	0.37	3.92
**PGSI score (wave 1)**			p=0.842
PGSI score	1.01	0.91	1.12
**Perceived loneliness (wave 1)**			p=0.943
Not at all/Not often/Sometimes	Reference		
Very much	1.03	0.46	2.32
**Impulsivity (wave 1)**			p=0.019
Impulsivity score (wave 1)	1.46	1.06	2.00
**Happiness (wave 1)**			p=0.011
Happiness scores (higher scores, higher happiness)	0.81	0.69	0.95
**Anxiousness (wave 1)**			p=0.101
Anxiousness score (higher scores, higher Anxiousness)	1.12	0.98	1.28
**Suicide attempts (wave 1)**			p<0.0001
No	Reference		
Yes	6.38	2.67	15.21
**Hosmer-Lemeshow Goodness of Fit**			
F-statistic (9, 1920)	1.47		
Prob>F	0.1548		

**Supplementary Table 2: Sensitivity testing for adjusted odds ratios for suicide attempts at wave 2: model 3, without prior suicide attempts**			
	AOR	95% CI (lower)	95% CI (upper)
**Change in Problem Gambling Severity Index (PGSI) Score**			p=0.006
No change in PGSI score	Reference		
PGSI scores increased	3.15	1.47	6.75
PGSI scores decreased	0.91	0.33	2.49
**PGSI score (wave 1)**			p=0.126
PGSI score	1.06	0.98	1.15
**Perceived loneliness (wave 1)**			p=0.189
Not at all/Not often/Sometimes	Reference		
Very much	1.55	0.81	2.98
**Impulsivity (wave 1)**			p=0.002
Impulsivity score (wave 1)	1.54	1.17	2.03
**Happiness (wave 1)**			p=0.022
Happiness scores (higher scores, higher happiness)	0.85	0.73	0.98
**Anxiousness (wave 1)**			p=0.010
Anxiousness score (higher scores, higher Anxiousness)	1.16	1.04	1.31
**Hosmer-Lemeshow Goodness of Fit**			
F-statistic (9, 1443)	0.29		
Prob>F	0.9788		

Appendix G: STROBE checklist

STROBE Statement—Checklist of items that should be included in reports of ***cohort studies***

	Item No	Recommendation	Location in manuscript
**Title and abstract**	1	(*a*) Indicate the study's design with a commonly used term in the title or the abstract	Title states online longitudinal survey
		(*b*) Provide in the abstract an informative and balanced summary of what was done and what was found	Summary
**Introduction**			
Background/rationale	2	Explain the scientific background and rationale for the investigation being reported	Background, paragraphs 1-4
Objectives	3	State specific objectives, including any prespecified hypotheses	Background, final paragraph
**Methods**			
Study design	4	Present key elements of study design early in the paper	Methods: study design and participants: paragraphs 1-3
Setting	5	Describe the setting, locations, and relevant dates, including periods of recruitment, exposure, follow-up, and data collection	Study design and participants
Participants	6	(*a*) Give the eligibility criteria, and the sources and methods of selection of participants. Describe methods of follow-up	Study design and participants
		(*b*) For matched studies, give matching criteria and number of exposed and unexposed	Not applicable
Variables	7	Clearly define all outcomes, exposures, predictors, potential confounders, and effect modifiers. Give diagnostic criteria, if applicable	Full section on measures
Data sources/ measurement	8*	For each variable of interest, give sources of data and details of methods of assessment (measurement). Describe comparability of assessment methods if there is more than one group	Full section on measures
Bias	9	Describe any efforts to address potential sources of bias	Study design and participants: paragraph 1
Study size	10	Explain how the study size was arrived at	Appendix D
Quantitative variables	11	Explain how quantitative variables were handled in the analyses. If applicable, describe which groupings were chosen and why	Full section on measures
Statistical methods	12	(*a*) Describe all statistical methods, including those used to control for confounding	Full section on Statistical Methods
		(*b*) Describe any methods used to examine subgroups and interactions	Not applicable
		(*c*) Explain how missing data were addressed	Appendix C
		(*d*) If applicable, explain how loss to follow-up was addressed	Study design and participants: paragraph 1; Appendix B
		(*e*) Describe any sensitivity analyses	Appendix F
**Results**			
Participants	13*	(a) Report numbers of individuals at each stage of study—eg numbers potentially eligible, examined for eligibility, confirmed eligible, included in the study, completing follow-up, and analysed	Study Design and Participants: paragraph 1.

		(b) Give reasons for non-participation at each stage	Reasons unknown
		(c) Consider use of a flow diagram	Not Applicable
Descriptive data	14*	(a) Give characteristics of study participants (eg demographic, clinical, social) and information on exposures and potential confounders	Table 2
		(b) Indicate number of participants with missing data for each variable of interest	Appendix C
		(c) Summarise follow-up time (eg, average and total amount)	Study design and participants, paragraph 1
Outcome data	15*	Report numbers of outcome events or summary measures over time	Table 1; results paragraph 1.
Main results	16	(*a*) Give unadjusted estimates and, if applicable, confounder-adjusted estimates and their precision (eg, 95% confidence interval). Make clear which confounders were adjusted for and why they were included	Tables 2 and 3; statistical analyses section
		(*b*) Report category boundaries when continuous variables were categorized	Not Applicable
		(*c*) If relevant, consider translating estimates of relative risk into absolute risk for a meaningful time period	Not Applicable
Other analyses	17	Report other analyses done—eg analyses of subgroups and interactions, and sensitivity analyses	Appendix F
**Discussion**			
Key results	18	Summarise key results with reference to study objectives	Discussion paragraph 1
Limitations	19	Discuss limitations of the study, taking into account sources of potential bias or imprecision. Discuss both direction and magnitude of any potential bias	Fully discussed in discussion; paragraph 5
Interpretation	20	Give a cautious overall interpretation of results considering objectives, limitations, multiplicity of analyses, results from similar studies, and other relevant evidence	Discussion, paragraphs 3-4
Generalisability	21	Discuss the generalisability (external validity) of the study results	Discussion – paragraph on limitation
**Other information**			
Funding	22	Give the source of funding and the role of the funders for the present study and, if applicable, for the original study on which the present article is based	Role of funding source

*Give information separately for exposed and unexposed groups.

Note: An Explanation and Elaboration article discusses each checklist item and gives methodological background and published examples of transparent reporting. The STROBE checklist is best used in conjunction with this article (freely available on the Web sites of PLoS Medicine at http://www.plosmedicine.org/, Annals of Internal Medicine at http://www.annals.org/, and Epidemiology at http://www.epidem.com/). Information on the STROBE Initiative is available at http://www.strobe-statement.org.
